# Your Money or Your Sense of Smell? A Comparative Analysis of the Sensory and Psychological Value of Olfaction

**DOI:** 10.3390/brainsci12030299

**Published:** 2022-02-23

**Authors:** Rachel S. Herz, Martha R. Bajec

**Affiliations:** 1 Department of Psychiatry and Human Behavior, Brown University Medical School, Providence, RI 02912, USA; 2 Department of Psychology and Neuroscience, Boston College, Chestnut Hill, MA 02467, USA; 3 HCD Research, Inc., Flemington, NJ 08822, USA; martha.bajec@hcdi.net

**Keywords:** olfaction, smell, vision, hearing, value, meaning, importance, life-stage, gender, commodities, COVID-19

## Abstract

In public discourse, the sense of smell is typically characterized as the least important of the five senses. However, there are very little empirical data on this topic. Recently, much more attention has been brought to the sense of smell since olfactory dysfunction is a primary and often long-term symptom of COVID-19 infection. It was therefore of interest to expand research on the perceived value of olfaction in the current cultural condition. We developed a survey that directly compared the value of the senses of smell, hearing, and vision with each other and in relation to nine common items representing digital, material, personal, and physical commodities of varying social and emotional meaningfulness (phone, $10,000, favorite social media, online shopping, favorite streaming service, dream vacation, pet, hair, little left toe). In total, four hundred and seven female and male respondents comprising two life-stage groups (college students, general public adults) participated in our online survey study during winter–spring of 2021. The results reveal that the sense of smell was perceived as vastly less important than vision and hearing and much less valuable than various common commodities. We also found that life-stage and gender mediated our findings. For example, one-quarter of the college student respondents would give up their sense of smell in order to keep their phone and nearly half of all women would give up their sense of smell to keep their hair. Our data further illustrate that the senses of vision and hearing are valued relatively similarly. A number of questions arise from the present data and suggestions for ways in which our survey can be expanded and altered to address further research are discussed.

## 1. Introduction

The sense of smell has been regarded as the least important of the five senses in western culture since at least the writings of Plato [1]. However, depending on the historical source, olfaction is sometimes displaced by taste or touch for the lowest rank. By contrast, vision reigns supreme in the Western tradition, and there is a large scientific literature in the domains of cognition and perception on “visual dominance”. Visual dominance refers not only to neural processing [2] but also to vision’s cultural and social primacy. For example, vision verbs dominate the conversation [3]. Notably, in several non-Western cultures, smell plays a major role in rituals and other activities [4,5,6,7,8]; for Andaman Islanders, scents are “vital energies”; and hunter-gatherer groups such as the Semaq Beri of the Malay peninsula have a more extensive and detailed vocabulary for olfactory attributes than they do for color hues [9]. It is also the case that the attention, value, and meaning placed on scent in Western culture has risen and fallen dramatically during different historical periods [5,10]. For instance, in early Christian rituals, scent enabled knowledge of the divine, and baptisms involved anointing the infant’s nostrils with scented oil to grant eternal “sweetening” [11]. Thus, the relative sensory value of olfaction is contextually mediated and not an absolute value of the sense [5,6].

Cross-cultural and historical accounts notwithstanding, the view that olfaction is the least important of the senses is witnessed in multiple domains of contemporary Western society. The American Medical Association Guides to Permanent Impairment [12] states that “loss of smell” is equal to 1–5% of a person’s earnings, while loss of vision is given 85% of an individual’s worth. Likewise, it has been shown that the average person does not give their sense of smell much value. In a large survey study on the importance and attention to olfaction in daily life, Wrzesniewski et al. [13] found that completely losing one’s sense of smell was ranked as equivalent to losing one’s little left toe or hearing in one ear. A direct examination of the relative importance of the senses was undertaken by Schifferstein [14]. In this study, which was focused on the importance of various sensory experiences in product usage, respondents also answered the question: “suppose you were asked to give up one of your senses and you would have the choice, which one would it be?” It was found that the rank ordering of the senses from which they “would miss the most” to which they “would miss the least” was vision, hearing, touch, taste, and smell. Apart from these reports, there are no investigations, that we know of, where the value of the sense of smell has been compared to the value of the other senses.

Notably, since the SARS-CoV-2 virus began circulating the globe in late 2019, there has been a dramatic increase in media attention and discussion of the sense of smell, as olfactory loss and dysfunction are diagnostic and often persistent symptoms of COVID-19 [15,16,17]. In addition to media reporting, spikes in Google searches for “smell loss” and related terms such as “anosmia” have been so prevalent that they have been used as diagnostic tools to track COVID-19 outbreaks [18,19]. Thus, it was of particular interest to assess the value of the sense of smell at this current moment in history. 

The goal of the present research was to compare the value of the sense of smell with the value of vision and hearing in the wake of the COVID-19 pandemic. To address this goal, we created a short survey that examined how olfaction was evaluated compared to the senses of vision and hearing. To obtain a deeper and more nuanced understanding of the relative importance of these senses, we further examined how they were valued in relation to various common commodities of differing social, physical, and emotional meaningfulness. For example, “would you rather lose your sense of smell/vision/hearing or your phone? Would you rather lose your sense of smell/vision/hearing or your pet? Would you rather lose your sense of smell/vision/hearing or your favorite streaming service?” Additionally, we performed a pseudoreplication of Wrzesniewski et al. [13] and included the commodity “little left toe”; “would you rather lose your sense of smell/vision/hearing or your little left toe?” 

Exposure to traditional news media and reckoning with physical decline both increase with age and life-stage [20,21]. We thus expected more mature participants to have both greater knowledge and exposure to the negative impact of smell loss (e.g., as a result of COVID-19) and higher general awareness of sensory and physical dysfunction. To address whether age/life experiences (life-stage) would affect the value placed on the senses (especially olfaction), individuals from two life-stage groups were recruited: (1) undergraduate college students and (2) adults from the general population. We also examined whether gender would mediate the findings, as females have been shown to be generally more engaged with scent in everyday life than males [22], and to have somewhat better olfactory acuity over their lifespan [23,24,25]. 

Our hypotheses were as follows: (1) Respondents would be more willing to give up their sense of smell than their senses of hearing or vision; (2) Individuals in the older, more life-experienced general public group would place a higher value on their sense of smell than college students; (3) Female respondents would be less willing to give up their sense of smell than male respondents; (4) As a pseudoreplication of Wrzesniewski et al. [13], respondents would be more willing to give up their sense of smell for their little left toe than they would be willing to give up vision or hearing for their little left toe. 

We also explored how the emotional value and meaningfulness of the commodity in question modulated responses and whether this varied across the three senses. For example, would more respondents be willing to give up a sense to keep their pets and phones than to keep their social media and streaming services? And would these effects be observed for all three senses or just smell and/or hearing but not vision? Thus, we were able to explore the discrepancy between the value placed on the sense of smell with the value placed on vision and hearing, as well as the relative value of hearing compared to vision. Our study provides the first report on how the senses of olfaction, hearing, and vision are comparatively valued and evaluated in relation to a range of personal commodities. 

## 2. Methods

### 2.1. Participants 

A total of 407 individuals completed the survey developed for this research. In total, ninety-six undergraduate students from Boston College and 92 undergraduates from Brown University comprised the college student group (CS), and 219 adults from the general public/population (GP) comprised the more mature life-stage group (see 3.1 *Group Characteristics* below and in Table 1 for details). College students were solicited from Facebook pages for the graduating years 2021–2024 at their respective institutions. Adults from the general public were solicited through the personal social media posts (Twitter, LinkedIn, Facebook) of colleagues, friends, and family of the authors who were not involved in chemosensory research. All solicitations asked for volunteers of 18 years and older to participate in a brief survey on the value of the senses and, if interested, to click on a link. No compensation or inducements were offered. The study was approved by the Boston College Institutional Review Board (9 February 2021; IRB Protocol Number: 21.216.01e), and the Qualtrics links to the survey for the Boston College undergraduates and the survey for the GP group began with a Boston College consent form. Brown University waived the necessity for Institutional Review Board approval. Nevertheless, the Qualtrics link for Brown University undergraduates also began with a consent form. 

### 2.2. Survey

A questionnaire was developed on Qualtrics (Provo, UT, USA) survey software in February 2021 to assess the perceived value of olfaction, hearing, and vision with each other and in relation to various commodities. During survey development, draft versions were discussed with colleagues and the survey was adjusted. Pilot testing with a small group of individuals confirmed the final version of the survey. 

The survey began by asking for demographic information pertaining to age, gender (female, male, non-binary, prefer not to say), income level (high, middle, low, prefer not to say), and any severe sensory impairments without correction in vision, hearing, or smell (participants could indicate impairment in more than one sense or none). A total of nine principal survey items followed that asked respondents to choose whether they would rather lose their sense of smell/hearing/vision or various social, emotional, and physical commodities. It was explained that in the shorthand of the survey “smell”, “hearing”, “vision” meant their ability to smell, their ability to hear, and their ability to see. A final two items on the survey asked respondents to indicate what “anosmia” meant from four options and to pick what they would least mind losing: their ability to smell, their ability to see, or their ability to hear. Our interest in the correct definition of “anosmia” was as a proxy for the level of awareness of olfactory dysfunction and to evaluate this in relation to differences between life-stage group and gender, as well as how this may translate to the value placed on the sense of smell. The full survey is presented in Appendix A.

### 2.3. Procedures

Interested volunteers who visited the webpage where the survey solicitation was posted clicked on a link that took them to the survey platform on Qualtrics. Volunteers first saw a consent form that briefly described the study. If the participant “agreed” to participate and responded with a number greater than or equal to 18 for their age, a webpage appeared with the survey items. The survey was compatible with smartphone and computer viewing formats and automatically loaded according to the participant’s device. Participants were able to skip/not respond to any item, to stop responding at any point, and there was no time limit. The average survey completion time was 3.72 min (SD = 6.03).

The survey link for the Boston College students was available between 17 February 2021 and 21 March 2021. The survey link for Brown University students was available between 5 March 2021 and 22 March 2021. The survey link for the GP group was posted on social media platforms beginning on 7 March 2021 and taken down on 7 May 2021. 

### 2.4. Data Analyses

Undergraduate respondents from the two institutions (Boston College and Brown University) were combined into one College Student (CS) group, as their data did not differ significantly on the sociodemographic variables assessed and were compared with the General Public/Population (GP) group for all analyses. In total, six individuals in the CS group and one in the GP group self-reported their gender as non-binary, and four respondents in the CS group and one in the GP group did not disclose their gender. Because there were too few respondents to support developing the gender categories of ‘non-binary’ and ‘non-reporting’, data from those who identified as non-binary or did not report their gender were omitted from the analyses involving gender; their data were included for other analyses. The data analyzed were from respondents who answered at least some of the demographic questions and at least one of the commodity questions (no one answered less than 45% of the commodity questions). Missing data were treated as such, and percentages were calculated based on the actual number of responses for that particular question. Percentages presented in the Figures are rounded for readability and are provided to two decimal places in the text. 

Age, income level, and gender were compared between the GP and CS groups and between male and female genders using the Chi-Square Statistic (χ2) and the Student’s t-test. The statistical significance of χ2 values for comparisons with a low number of respondents (indicated in the Figures by ‘/’ before significance symbols) was confirmed by Yate’s correction and Fisher’s exact test [26]. Univariate analyses were conducted for “Hypothesis Testing” to ascertain the impact of the variables under consideration (i.e., sense, commodity, group, gender) for willingness to give up a sense. To determine which variables (i.e., sense, commodity, group, gender) and their interactions contributed to the willingness to give up a sense, model building (see below) was based on the outcomes of the univariate comparisons conducted as a screening step. Due to the novelty of the present research, which made the avoidance of both false-negative and false-positive outcomes a consideration and the interdependence of the variables examined through the planned comparisons undertaken, corrections were not made for the multiple comparisons conducted with univariate statistics [27,28,29,30,31]. All statistical analyses are reported as significant where *p* < 0.05.

Generalized linear mixed models (GLMM) with binomial distribution and logit link functions were used to explore all comparisons (including within categories, i.e., GP vs. CS; female vs. male) and their interactions. For all GLMMs, respondent intercepts were included as random effects as they were repeated measures for each respondent [32] while all other variables were treated as fixed effects. For sense and commodity, hearing and $10,000 (10k) were used as the reference categories. For group and sex, GP and female were used as the references. Although both Bayesian Information Criterion (BIC) and Akaike Information Criterion (AIC) were considered as measures of model fit, the aim of modeling in the current research was to obtain the most concise and least complex prediction of willingness to give up a sense; therefore, BIC was primarily used [33,34]. Marginal predicted means from GLMM odds ratios were used to obtain the probabilities for giving up a sense. Descriptive and univariate statistical analyses were conducted using XLSTAT 2021.4.1 (Addinsoft, New York, NY, USA) software. GLMMs and associated analyses were conducted using Stata 16 (StataCorp, College Station, TX, USA) software.

## 3. Results

### 3.1. Group Characteristics

Characteristics of the GP and CS groups are detailed in Table 1. The GP group (*M* age = 47.58, *SD* = 12.94) was significantly older than the CS group (*M* age = 22.20, *SD* = 8.72); *t* = 22.82, *p* < 0.0001, and included significantly more middle income (GP = 64.84% vs. CS = 53.30%) and significantly fewer high income (GP = 21.92% vs. CS = 32.97%) individuals, χ^2^(1, *N* = 407) = 6.80, *p* = 0.03. No significant difference was observed for gender between GP and CS groups with females comprising 77.63% and 76.06% of each group, respectively, χ^2^(1, *N* = 407) = 7.14, *p* = 0.07. The majority of respondents in both the GP and CS groups reported either no sensory impairment (GP = 71.23%, CS = 75.53%) or an impairment in vision (GP = 23.74%, CS = 20.74%), and there were no significant differences between the groups for these two impairment categories, χ^2^(1, *N* = 407) = 0.64, *p* = 0.4. 

### 3.2. Hypothesis Testing

**Hypothesis** **1** **(H1).** *Respondents would be more willing to give up their sense of smell than their senses of hearing or vision*.

This hypothesis was tested in three ways. First, by analysis of the forced-choice question that explicitly asked which one of the three senses they would give up; second, by analyzing willingness to give up each sense collapsed across the responses for all commodities; third, by analyzing willingness to give up a sense for each commodity. All analyses showed that smell was given up much more than hearing and vision. 

**Figure 1:**
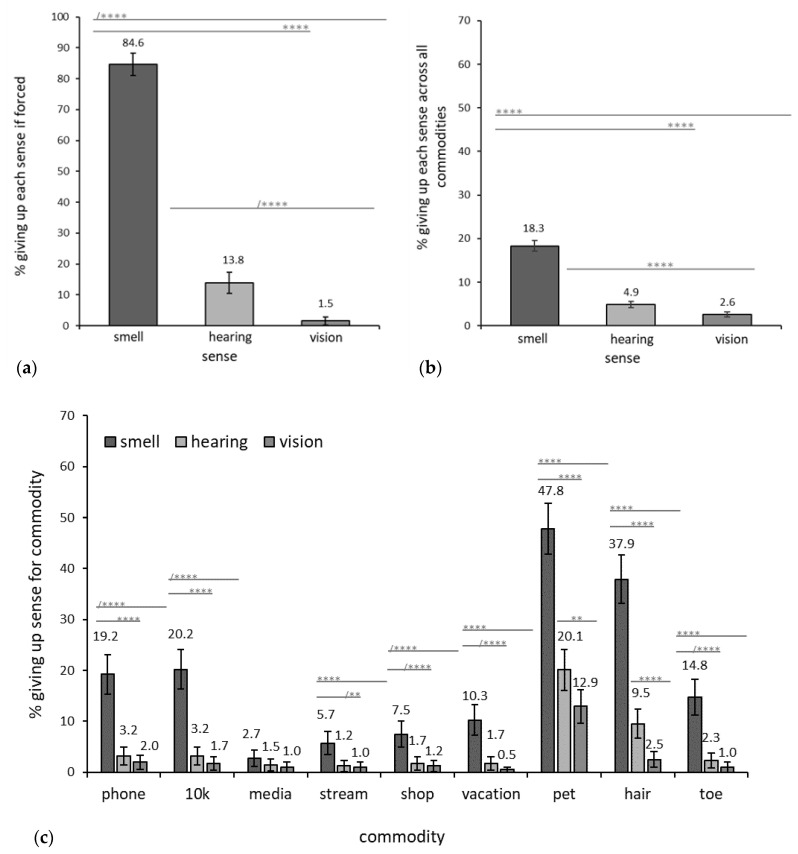
Willingness to give up each sense across all respondents by (**a**) forced choice between the three senses, (**b**) responses by sense collapsed over all commodities, and (**c**) responses by sense and commodity. Note: significance indicated by, ** *p <* 0.01, **** *p* < 0.0001; “/” indicates caution on interpretation due to low number of responses of one of the comparators (for details see Methods section).

When asked explicitly to select a sense to give up, smell (84.62%) was selected by significantly more respondents than either hearing (13.85%), χ^2^(1, *N* = 390) = 113.90, p < 0.0001 or vision (1.54%), χ^2^(1, *N* = 390) = 193.47, *p* < 0.0001. See Figure 1a. When responses for willingness to give up the senses were collapsed across commodities, smell (18.32%) was given up significantly more than either hearing (4.88%), χ^2^(1, *N* = 407) = 317.87, *p* < 0.0001) or vision (2.61%), χ^2^(1, *N* = 407) = 474.04, *p* < 0.0001). See Figure 1b.

When willingness to give up a sense was compared against the specific commodities, smell was given up significantly more than either hearing or vision for **phone** [smell = 19.21% vs. hearing = 3.19%, χ^2^(1, *N* = 407) = 50.86, *p* < 0.000001; vs. vision = 1.97%; χ^2^(1, *N* = 407) = 63.73, *p* < 0.000001)], **10k** [smell = 20.20% vs. hearing = 3.19%, χ^2^(1, *N* = 407) = 56.94, *p* < 0.000001; vs. vision = 1.72% χ^2^(1, *N* = 407) = 70.98, *p < 0.000001*)], **dream vacation** [smell = 10.25% vs. hearing = 1.76%, χ^2^(1, *N* = 407) = 25.71, *p* < 0.000001; vs. vision = 0.50%, χ^2^(1, *N* = 407) = 37.38, *p* < 0.000001)], **pet** [smell = 47.82% vs. hearing = 20.10%, χ^2^(1, *N* = 407) = 66.50, *p <* 0.000001; vs. vision = 12.92%, χ^2^(1, *N* = 407) = 111.62, *p* < 0.000001)], **hair** [smell = 37.94% vs. hearing = 9.55%, χ^2^(1, *N* = 407) = 88.60, *p* < 0.000001; vs. vision = 2.52%, χ^2^(1, *N* = 407) = 154.40, *p < 0.000001*)], **little left toe** [smell = 14.76% vs. hearing = 2.28%, χ^2^(1, *N* = 407) = 39.31, *p < 0.000001*); vs. vision = 1.02%, χ^2^(1, *N* = 407) = 51.20, *p* < 0.000001)], **streaming service** [smell = 5.71% vs. hearing = 1.24%,(χ^2^(1, *N* = 407) = 12.03, *p* = 0.001); vs. vision = 0.99%, χ^2^(1, *N* = 407) = 13.83, *p* = 0.0002] and **online shopping** [smell = 7.46% vs. hearing = 1.73%, χ^2^(1, *N* = 407) = 15.11, *p* = 0.0001); vs. vision = 1.24%, χ^2^(1, *N* = 407) = 18.67, *p =* 0.00002)]. See Figure 1c. Smell (2.72%) was given up least often for **social media**, and for this commodity only did not differ significantly from hearing (1.48%), χ^2^(1, *N* = 407) = 1.50, *p* = 0.22, or vision (0.99%), χ^2^(1, *N* = 407) = 3.31, *p* = 0.07.

**Hypothesis** **2** **(H2).** *Individuals in the older more experienced general population (GP) group would place higher value on their sense of smell than college students (CS)*.

**Figure 2:**
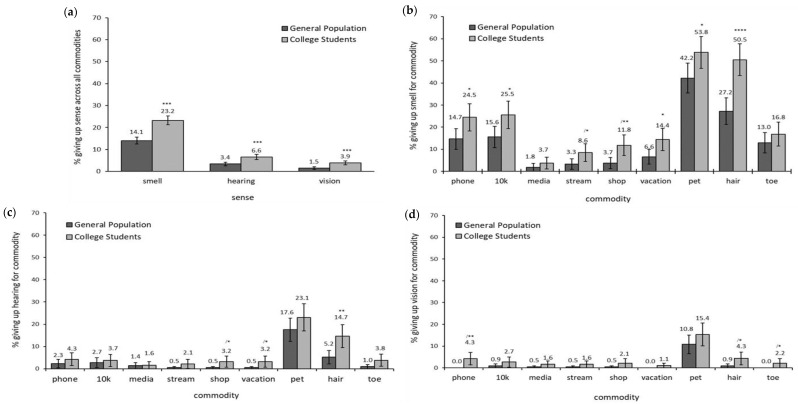
Willingness to give up each sense between the General Population and College Student groups (**a**) by responses collapsed over all commodities for each sense (**b**) by responses to each commodity compared to smell, (**c**) by responses to each commodity compared to smell, (**d**) by responses to each commodity compared to vision. Note: significance indicated by * *p <* 0.05, ** *p <* 0.01, *** *p <* 0.001, **** *p <* 0.0001; / indicates caution on interpretation due to low number of responses of one of the comparators (for details see Methods section).

This hypothesis was strongly confirmed. As can be seen in Figure 2a, significantly more respondents in the CS group (23.24%) gave up their sense of smell than in the GP group (14.06%), χ^2^(1, *N* = 407) = 50.47, *p* < 0.0001. Significantly more respondents in the CS group also gave up hearing (6.57%) and vision (3.89%) than in the GP group (hearing = 3.41%, χ^2^(1, *N* = 407) = 19.22, *p* < 0.0001; vision = 1.51%, χ^2^(1, *N* = 407) = 19.93, *p* < 0.0001). 

The GP and CS groups were further compared for their willingness to give up their senses of smell, hearing, and vision relative to giving up each specific commodity. Here it was found that significantly more respondents in the CS group gave up their sense of smell than respondents in the GP group for **phone** [GP = 14.68% vs. CS = 24.47%; χ^2^(1, *N* = 407) = 6.23, *p* = 0.01], **10k** [GP = 15.60% vs. CS = 25.53%; χ^2^(1, *N* = 407) = 6.18, *p* = 0.01], **streaming service** [GP = 3.26% vs. CS = 8.56%; χ^2^(1, *N* = 407) = 5.21, *p* = 0.02], **online shopping** [GP = 3.72% vs. CS = 11.83%, χ^2^(1, *N* = 407) = 9.47, *p* = 0.002], **dream vacation** [GP = 6.60% vs. CS = 14.44%; χ^2^(1, *N* = 407) = 6.62, *p* = 0.01], **pet** [GP = 42.23% vs. CS = 53.85%, χ^2^(1, *N* = 407) = 5.22, *p* = 0.02], and **hair** [GP = 27.23% vs. CS = 50.54%, χ^2^(1, *N* = 407) = 22.77, *p* < 0.0001]. There were no significant differences between groups for willingness to give up smell in favor of **social media** and **little left toe**. See Figure 2b. 

The CS group was also significantly more willing than the GP group to give up their sense of hearing for **online shopping** [GP = 0.46% vs. CS = 3.21%; χ^2^(1, *N* = 407) = 4.43, *p* = 0.04], **dream vacation** [GP = 0.47% vs. CS = 3.21%, χ^2^(1, *N* = 407) = 4.35, *p* = 0.04], and **hair** [GP = 5.16% vs. CS = 14.67%, χ^2^(1, *N* = 407) = 10.31, *p* = 0.001). See Figure 2c. Significant differences were further observed between the GP and CS groups for giving up their sense of vision for their **phone** [GP = 0% vs. CS = 4.26%, χ^2^(1, *N* = 407) = 9.46, *p* = 0.002)], **hair** [GP = 0.94% vs. CS = 4.35%, χ^2^(1, *N* = 407) = 4.64, *p* = 0.03], and **little left toe** [GP = 0% vs. CS = 2.17%, χ^2^(1, *N* = 407) = 4.59, *p* = 0.03]. See Figure 2d.

**Hypothesis** **3** **(H3).** *Female respondents would be less willing to give up their sense of smell than male respondents*.

**Figure 3:**
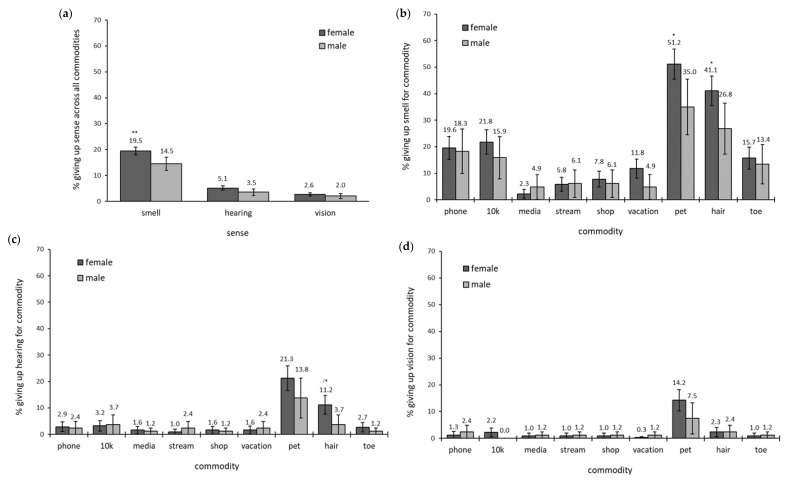
Willingness to give up each sense between Female and Male gender groups (**a**) by responses collapsed over all commodities for each sense, (**b**) by responses to each commodity compared to smell, (**c**) by responses to each commodity compared to hearing, (**d**) by responses to each commodity compared to vision. Note: significance indicated by * *p <* 0.05, ** *p <* 0.01; / indicates caution on interpretation due to low number of responses of one of the comparators (for details see Methods section).

Contrary to our hypothesis, when collapsed across all commodities, more females (19.51%) than males (14.54%) were willing to give up their sense of smell, χ^2^(1, *N* = 395) = 9.53; *p* = 0.002. However, the number of respondents who were willing to give up hearing [females = 5.14% vs. males = 3.53%, χ^2^(1, *N* = 395) = 3.28, *p* = 0.07] and vision [females = 2.65% vs. males = 2.04%, χ^2^(1, *N* = 395) = 0.88, *p* = 0.4] did not differ significantly between genders (Figure 3a). 

To further explore the gender effect on smell, the willingness to give up each sense against each commodity was assessed. The only commodities that differed significantly between males and females was the willingness to give up smell in favor of keeping their **pet** [females = 51.18% vs. males = 35.00%, χ^2^(1, *N* = 395) = 6.61, *p* = 0.01] and **hair** [females = 41.12% vs. males = 26.83%, χ^2^(1, *N* = 395) = 5.59, *p* = 0.02]. See Figure 3b. Females (11.18%) also gave up hearing in favor of their **hair** significantly more than males (3.66%), χ^2^(1, *N* = 395) = 4.22, *p* = 0.04 (see Figure 3c). No significant differences were observed between females and males for the willingness to give up vision for any commodity (see Figure 3d). 

Since only the commodities of pet and hair differed significantly between genders, the analysis collapsed across commodities was recalculated without pet and hair. Doing so rendered the gender effect for smell nonsignificant [females = 12.10% vs. males = 9.93%, χ^2^(1, *N* = 395) = 2.08, *p* = 0.15]. Gender differences for hearing and vision remained nonsignificant.

**Hypothesis** **4** **(H4).** *Respondents would be more willing to give up their sense of smell for their **little left toe** than they would be willing to give up vision or hearing for their little left toe*.

This hypothesis was confirmed. Significantly more respondents were willing to give up smell (14.76%) for their little left toe than either their senses of hearing (2.28%), χ^2^(1, *N* = 407) = 39.31, *p* < 0.000001, or vision (1.02%), χ^2^(1, *N* = 407) = 51.20, *p* < 0.000001. Hearing and vision did not differ significantly for how often they were given up for their little left toe, χ^2^(1, *N* = 407) = 1.96, *p* = 0.16. Figure 1c shows the effect of sense on giving up one’s little left toe. 

### 3.3. Modelling Willingness to Give up the Senses

**Figure 4:**
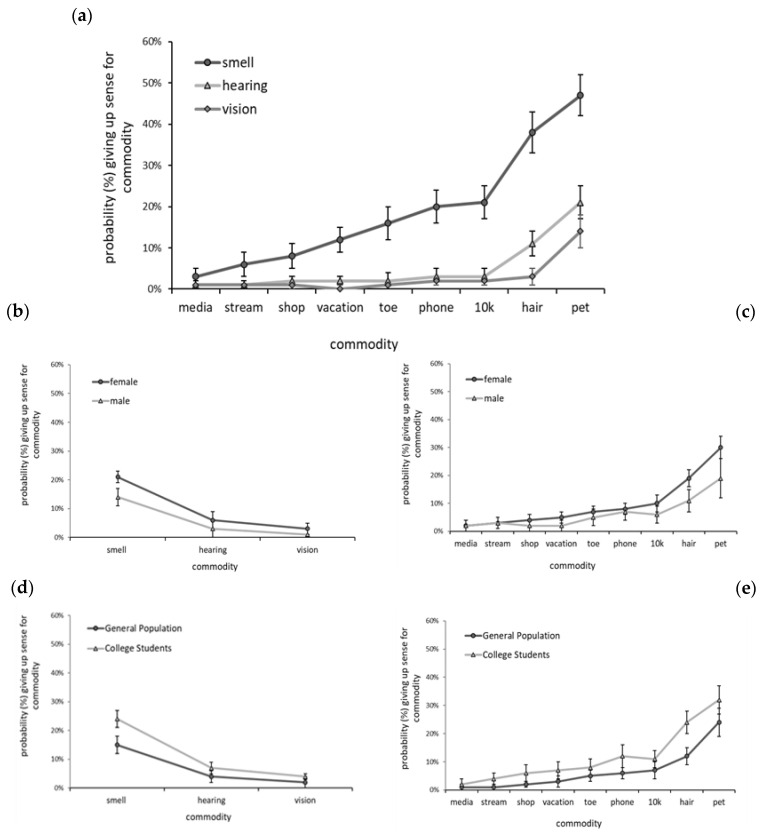
Probability of giving up senses (**a**) across all respondents by sense and commodity, (**b**) collapsed across commodities by sense for genders, (**c**) collapsed across senses by commodity for genders, (**d**) collapsed across commodities by sense for general population and college student groups, and (**e**) collapsed across senses by commodity for general population and college student groups.

The objective of the modeling conducted for the present research was to understand the contribution of the variables under investigation (sense, commodity, group, gender) on the decision to give up smell, hearing, and vision. Toward this end, the most concise and least complex model was preferred; therefore, the Bayesian Information Criterion (BIC), along with each parameter’s significance in the model, was used to evaluate the model’s fit. Marginal predicted means were used to obtain probabilities of giving up each sense by each commodity (Figure 4a), as well as gender by sense and by commodity (Figure 4b,c), and group by sense and by commodity (Figure 4d,e). 

Based on the significant findings from the univariate analyses, an initial model was evaluated by GLMM (Model 1) which included all variables of interest (i.e., sense, commodity, group, and gender) as well as their 2-way interactions (i.e., sense*commodity, commodity*sample, commodity*gender, sense*sample, and sense*gender; see Table 2). Significant main effects were observed for the variables sense, χ^2^(2, *N* = 407) = 137.08, *p* < 0.00001, commodity, χ^2^(8, *N* = 407) = 236.84, *p* < 0.00001, and group, χ^2^(1, *N* = 407) = 12.35, *p* = 0.0004), but not gender, χ^2^(1, *N* = 407) = 2.17, *p* = 0.1). The only interaction to reach significance in Model 1 was sense*commodity, χ^2^(16, *N* = 407) = 59.08, *p* < 0.00001. Additional models were explored to examine potential relationships between the key variables (data not shown). Model 2, which had the lowest BIC (4009), was the best fitting model that included all the variables of interest, sense, χ^2^(2, *N* = 407) = 208.38, *p* < 0.00001, commodity, χ^2^(8, *N* = 407) = 314.31, *p* < 0.00001, group, χ^2^(1, *N* = 407) = 20.80, *p* = 0.00001), and gender, χ^2^(1, *N* = 407) = 6.70, *p* < 0.01). 

Given the observed impact of **pet** and **hair** on willingness to give up a sense, they were removed from the commodity list, and model-building was conducted again. The full model without **pet** and **hair** (Model 3), where main effects of sense, χ^2^(2, *N* = 407) = 69.91, *p* < 0.00001, commodity, χ^2^(6, *N* = 407) = 22.53, *p* < 0.01, group, χ^2^(1, *N* = 407) = 11.61, *p* = 0.001) and the sense*commodity interaction, χ^2^(12, *N* = 407) = 34.04, *p* < 0.001, were significant but gender and all other interactions were not, was used for further comparison of model fit (BIC = 2330). The final model (Model 4), which had the lowest BIC (2193), included only sense, χ^2^(2, *N* = 407) = 137.08, *p* < 0.00001, commodity, χ^2^(6, *N* = 407) = 33.57, *p* < 0.00001, group, χ^2^(1, *N* = 407) = 17.70, *p* < 0.00001), and the sense*commodity interaction, χ^2^(12, *N* = 407) = 17.70, *p* < 0.00001) as they were the only significant contributors to the model for willingness to give up senses (see Table 2). Ultimately, Model 4 was the most concise and least complex model, which was a key goal of the modelling conducted here, and it demonstrated the importance of **pet** and **hair** to respondents, especially females as their removal from the commodity list mitigated the impact of gender on willingness to give up senses. 

### 3.4. Exploratory Analyses of the Commodities, Relative Value of Hearing vs. Vision, and Knowledge of “Anosmia”

Commodities: Of the commodities evaluated here, pet and hair had the greatest emotional value, as respondents were most willing to give up any of their three senses for them. Almost half of all respondents (47.82%) were willing to give up their sense of smell for their **pet** and a fifth (20.10%) of respondents were willing to give up hearing to keep their pet. Perhaps the greatest indicator of the pet’s meaningfulness was the observation that 12.92% of respondents were willing to give up their vision in order to keep their pet (Figure 1c). **Hair** was also very meaningful, as almost 40% (37.94%) of all respondents were willing to give up smell to keep it, and 9.55% were willing to give up hearing. Only 2.52%, however, were willing to give up vision for their **hair**. GLMM results supported the findings summarized here; when controlling for other key variables, the highest probabilities for giving up any of the three senses were for hair and pet, as seen in Figure 4a. Indeed, the probabilities of giving up hearing and vision were similarly low for all commodities except hair [hearing = 11% vs. vision = 3%] and pet [hearing = 21% vs. vision = 14%], where the probabilities of giving up hearing were 3.5 times and 1.5 times greater, respectively, than they were for giving up vision. 

Only for the commodity **social media** were there no significant differences between willingness to give up smell, hearing, and vision, and all senses were given up at very low levels; 2.72%, 1.48%, and 0.99%, respectively. In all other cases, more respondents gave up their sense of smell to keep any commodity than they did for hearing or vision (Figure 1c). 

Hearing vs. Vision: Our survey allowed us to compare the relative value of hearing and vision and assess how differences in the value of these two senses were manifested. When asked explicitly to select a sense to give up, vision was given up by significantly fewer respondents (1.54%) than hearing (13.82%), χ^2^(1, *N* = 390) = 33.60, *p < 0.0001*. See Figure 1a. Additionally, when responses for willingness to give up the senses were collapsed across commodities, vision (2.61%) was given up by slightly but statistically fewer respondents than hearing (4.88%), χ^2^(1, *N* = 407) = 25.66, *p < 0.0001*. See Figure 1b. However, when responses were examined by the specific commodities, hearing and vision were given up at equivalently low frequencies in almost every case (see Figure 1c). No significant differences were observed between willingness to give up hearing and vision when contrasted against **phone**, **social media**, **streaming service**, **online shopping**, **dream vacation**, and **little left toe**. Only against **pet** (χ^2^(1, *N* = 407) = 31.08, *p* = < 0.007) and **hair** (χ^2^(1, *N* = 407) = 18.52, *p* = 0.00003) was hearing given up significantly more often than vision. When controlling for other key variables of interest via GLMM, the conclusion that vision and hearing were both given up at low levels was supported, as their probabilities of being given up were similarly low. See Figure 4a. 

Knowledge of “Anosmia”: Over all respondents, the vast majority (81.39%) correctly indicated that the definition of anosmia referred to the “inability to smell”. Life-stage and gender also mediated these findings. Significantly more respondents in the GP group (88.12%) than in the CS group (70.18%) correctly identified “anosmia”, χ^2^(1, *N* = 316) = 9.75, *p* = 0.002; as did significantly more females (84.81%) than males (73.61%), χ^2^(1, *N* = 309) = 4.73, *p* = 0.03.

## 4. Discussion

The results of our survey research dramatically illustrate the negligible value people place on their sense of smell and reveal a number of intriguing factors that support and mediate these findings. Consistent with previous empirical work [13,14] and contemporary Western designations (American Medical Association [12], hypothesis 1 was very strongly supported; respondents were much more willing to give up their sense of smell than their senses of hearing or vision. When directly asked to select one sense to give up, the vast majority of respondents chose to give up their sense of smell, in comparison to those who opted to give up hearing or vision. The sense of smell was also given up by far more respondents in favor of keeping the various commodities assessed than hearing or vision were (discussed further below). Thus, in spite of the COVID-19 pandemic directing a high level of public attention on the sense of smell, and the toll on the quality of life that anosmia takes [35,36], it appears that, as Schifferstein found in 2006 [14], olfaction remains ranked as the least valuable sense. It would be ideal if we could directly compare our data to Schifferstein [14] as a gauge for whether or how the value placed on the sense of smell has changed with time. However, as the importance of the sensory modalities in Schifferstein [14] was presented as rank averages it is not possible to convert our data to enable a meaningful comparison. In future studies, it is hoped that our findings may be a useful benchmark.

Hypothesis 2 was also clearly supported. When directly asked to give up one sense, respondents in the more mature GP group were less willing to give up their sense of smell than respondents in the CS group. Additionally, across commodities, the GP group was less willing to give up their sense of smell than the CS group. However, several interesting commodity-specific effects were observed (discussed below). 

We surmised that the reason respondents in the GP group were less inclined to give up their sense of smell than those in the CS group was because, being older and more life-experienced, GP respondents had a generally higher comprehension of physical/sensory impairment and more exposure to olfactory health information. We did not have a direct measure to confirm that the GP group was more informed about olfactory health; however, substantially more respondents in the GP knew the correct definition of “anosmia” than respondents in the CS, which implies greater olfactory health awareness. However, it may not be the case that the GP group was more aware of sensory impairment, as the percentage of respondents who reported an olfactory sensory impairment was equivalent between the two groups. Nonetheless, respondents in the GP group likely had a greater comprehension of physical decline as awareness of mortality is known to increase with age [37]. Another possibility for the difference between groups, based on the demographic data we collected, is that socio-economics were involved. The GP respondents were predominantly middle-class while there were proportionally more high-income respondents in the CS group. That said, wealth fails to account for the fact that the CS group was more willing to give up their sense of smell for the material commodities of their phone, $10,000, dream vacations, online shopping, and streaming services than respondents in the GP group were. 

Other explanations that were not captured by the brief demographic questions in our survey may also mediate the discrepancy in willingness to give up their sense of smell (as well as hearing and vision) between the GP and CS respondents. One possibility is the general education level. Although all the respondents in the CS group were engaged in higher education, we did not ask respondents to indicate their education level and it is certain that some proportion of respondents in the GP group had advanced degrees. Thus, general education level may be a mediator of the effects. Moving forward, a deeper exploration of individual differences that are involved in attachment to the sense of smell, as well as one’s senses in general, should be undertaken. 

Surprisingly, despite prior research showing greater female olfactory acuity and engagement [22,25], and that more female respondents in our study knew the correct definition of “anosmia”, which we construed as an indirect metric for understanding smell loss, we did not observe that females would be less willing to give up their sense of smell than males. Thus, hypothesis 3 was not supported. Rather, when all commodities were included in the analysis, we found the reverse; females were more likely to give up their sense of smell than males were. 

However, a closer examination of responses to the commodities revealed that the lower value placed on the sense of smell by women in our study was due to their placing an especially high value on their pets and hair compared to men. When the commodities of pet and hair were removed from the analysis no gender differences in willingness to give up the sense of smell were observed. We also found that gender was not a significant factor in our model building. Notably, there were approximately three times more female than male respondents, which should have skewed our findings towards finding a gender effect in favor of female responding if one existed. 

The null effect of gender is consistent with Wrzesniewski et al. [13] who found that the importance of olfaction was judged to be largely similar by men and women across a wide range of issues. Schifferstein [14] found a general effect of females giving higher value to all the senses than males, but smell did not stand out differently from vision, hearing, touch, or taste. Furthermore, although Oleszkiewicz et al. [22] reported that women reported more olfactory encounters on a daily basis than men, this finding was paradoxically driven by women in the group of participants who had olfactory deficits and was not found among healthy controls. Our findings contrast with physiological evidence for greater olfactory sensitivity among females than males [23,24,25]; however, physiological acuity may not mediate subjective responses. On balance, it seems that how questions are posed and what comparisons are made mediates whether gender will modify subjective engagement with the sense of smell. 

Substantiating the trivial value given to the sense of smell described by Wrzesniewski et al. [13], we found that respondents were much more willing to give up their sense of smell for their “little left toe” than they were willing to give up vision or hearing for their little left toe. Thus hypothesis 4 was supported. Notably, Wrzesniewski et al. [13] reported that permanent loss of smell was ranked on par with losing one’s little left toe. By contrast, smell fared better in our survey as we found that the majority of respondents were unwilling to trade their sense of smell for their little left toe. Various explanations for the difference in findings between these two studies are possible, not least of which is how the question was worded. However, from the totality of our results, we do not believe that the status of the sense of smell has been elevated in public opinion overall. 

As expected, barely any of the respondents were willing to give up hearing or vision in favor of their little left toe. Interestingly, the proportion of respondents willing to give up their sense of smell for their little left toe was equivalent between genders and life-stage groups, suggesting that the value of one’s toe is not meditated by demographic characteristics, at least not those evaluated here. The responses obtained for “little left toe” now lead to a discussion of some of the most interesting findings of our study: how the specific commodities in question mediated the results. 

In a shocking indictment of how little value is given to the sense of smell, especially among younger adults, a quarter of the college students surveyed stated that they would give up their sense of smell in order to keep their “phone”—an easily replaceable commodity. Just over a quarter of the CS respondents would also give up their sense of smell for “$10,000”, indicating that when it comes to one’s money or sense of smell, young adults are likely to take the money and run. In the GP group, relatively fewer respondents jettisoned their sense of smell for these commodities; however, the numbers were still surprisingly high; 15% and 16% of respondents, respectively, stated that they would give up their sense of smell in order to keep their “phone” and “$10,000”. 

In addition to revealing how easily the sense of smell was discarded in favor of simple material commodities (for example, 7% and 6%, respectively, of all respondents would give up their sense of smell to shop on Amazon or watch Netflix), several noteworthy patterns emerged. Of particular interest were the responses observed for “pet” and “hair”. The greatest willingness over any sense in favor of keeping a commodity was seen for one’s pet. Nearly half of all respondents would give up their sense of smell to keep their pet, and far more respondents were willing to give up hearing and vision in order to keep their pet compared to any other commodity. The salience of one’s pet is particularly striking if one considers that 13% of respondents would rather become blind than give up their pet. However, it may not be so startling if one considers that in contemporary North American culture most people confer family member status to their pets [38], substantial and persistent grief over the loss of a pet is often seen [39], and, in 2020, 103.6 billion dollars were spent on pets in the US alone [40], which exceeds the gross domestic product of a number of nations. 

The second most cherished commodity was “hair”, with over one-third of all respondents willing to part with their sense of smell in order to keep their hair, and 10% of respondents willing to give up their hearing to keep their hair. However, very few participants would give up vision to keep their hair. Premature hair loss is known to cause psychological distress [41]. However, for both the senses of smell and hearing marked gender differences in willingness to give up one’s hair were observed. Many more females were willing to part with the sense in question in order to keep their hair; 41% of female respondents were willing to part with their sense of smell to keep their hair vs. 27% of male respondents, and 11% of female respondents vs. 4% male respondents would give up hearing for their hair. This gender difference is not unexpected, given that femininity is highly linked to having a full head of hair [42]. Hair loss has also been shown to be associated with substantial psychological distress and decreased quality of life for women [43,44], and while men with baldness also experience stress it is typically less psychologically taxing for males due to societal norms associated with male hair loss [45]. Nevertheless, it is stunning evidence of the insignificance of smell that nearly half of all female respondents would give up this sensory system—a primary physiological, neurological, and psychological mode through which the world is experienced—in order to keep a physical feature with minimal functional utility. Notably, we did not specify the meaning of “hair”, and though it was assumed that most people interpreted “hair” as the hair on one’s head, it is possible that some respondents interpreted it as body hair, and/or facial hair, or any hair on the body/head. In future administrations of this survey, “hair” should be defined more precisely.

Female respondents were also more willing than males to give up their sense of smell for their pets. However, no gender differences were found for giving up the senses of hearing or vision for pets. Females have been shown to be more attached to companion animals than males [46] and to suffer more grief from pet death than males [39]. That no gender differences were observed for the senses of hearing and vision underscores how valuable these senses were perceived to be compared to smell, and that the importance of pets overall is very high. 

Relative to the sense of smell, but not hearing or vision, the desire to keep one’s “pet” and “hair” also distinguished the GP and CS groups. Substantially more CS than GP respondents would give up their sense of smell in order to keep their pets and hair. That college students were more attached to their pets is not unexpected since the CS respondents may have been away from home for the first time and loneliness increases bonding with pets [47], and pets provide emotional support and companionship [48]. The differences between the life-stage groups for the importance of hair were also likely driven by the excessive social value placed on the importance of physical appearance for young women and the stressors regarding physical attraction that younger individuals face [43,49]. It would now be important to know how life-stage mediates the value of other highly emotionally personal commodities. 

Our survey revealed that the rank order of commodities from most to least importance (by how willing respondents were to give up a sense for it, particularly the sense of smell) was pet, hair, $10,000, phone, little left toe, dream vacation, online shopping, favorite streaming service, and favorite social media site. However, when considering the types of commodities we included in our survey (digital, material, emotional, physical), it is intriguing that they did not cluster in an obvious way. That is, hair and little left toe (physical commodities) were valued quite differently, as were the material commodities of $10,000 and dream vacation, even though a dream vacation could easily cost $10,000. This suggests that how the commodities were interpreted is not straightforward. For example, a dream vacation may connote an aspirational goal while $10,000 connotes concrete wealth. It is also notable that the digital commodities of “online shopping” and “streaming service” were very similarly valued and yet “social media” was deemed far less important. Indeed, “social media” was the least valued of all the commodities surveyed, and the only commodity for which respondents were not more likely to give up their ability to smell over their ability to see. The very low value given to social media is especially surprising given the extensive literature detailing the obsession and addiction to social media in contemporary society [50,51,52]. It is possible that since modes of popular social media change so frequently and the content of what is trending is so transient and variable that the general concept of social media was not given much value. Thus, it may have been more meaningful had the commodity been phrased with greater specificity such as “your Tik Tok account”. On the other hand, it is possible that this survey item tapped into the despair that a preoccupation with social media incurs and that there is in fact a strong desire to spend less of one’s time on it [53]. In further examinations, the types of commodities analyzed should be expanded to discover what schemas underlie them.

With regard to the comparative value of hearing versus vision, our survey revealed that they were assessed much more similarly than might have been expected. When forced to choose a sense to give up there was a noticeable difference in the number of respondents willing to part with hearing versus sight; however, this difference was far less dramatic than the difference observed between the number of respondents giving up smell versus hearing. When respondent responses were collapsed across commodities for willingness to give up the sense, the difference between hearing and vision, though statistically meaningful, was quite small, and when the commodities were examined individually, the only commodities for which willingness to give up hearing and vision differed was for pet and hair; the most emotionally reactive commodities in our survey. Schifferstein [14] found that when participants were asked what sensory modality they would miss most if lost, hearing directly followed vision, and smell came in last of the five senses. However, when participants were asked to evaluate the importance of each of the senses in various product categories ranging from vehicles to personal care, the value of hearing fell considerably in importance [14]. This illustrates that context is critical when considering the value of the senses.

Prior to the COVID-19 pandemic, the term “anosmia” was not commonly known to non-clinicians. However, with loss of smell being a major symptom of COVID-19, we expected the term “anosmia” to have become familiar to average individuals. “Anosmia” rose dramatically as a search term during the pandemic [18], and was discussed in detail in a very widely disseminated and cited *New York Times* article [54]. Indeed, we found very high knowledge of the definition of “anosmia”, with over three-quarters of the respondents knowing the correct meaning. This provides indirect support for the supposition that COVID-19 has increased knowledge of smell loss and its terminology and suggests that “anosmia” has entered the common lexicon. 

We also observed that more respondents in the GP group knew the correct definition of “anosmia” and they also placed more value on their sense of smell than respondents in the CS group. We considered knowledge of “anosmia” to be a proxy for knowledge about olfactory dysfunction. Thus, we interpret this finding for the life-stage group as reflecting that GP respondents knew more about the negative impact on quality of life that anosmia can have, and this related to their valuing their sense of smell more highly than respondents in the CS group. We also observed that more females knew the correct definition of anosmia than did males. However, gender did not mediate our findings. It is noted that, due to the gender imbalance in our study, we do not know if knowledge of the correct meaning of “anosmia” has a bearing on the potential to find gender differences in appreciation for the sense of smell in future research. 

### Limitations 

There were several limitations to our study. First, for a comprehensive analysis of olfaction’s place among the senses, touch and taste should have been assessed. We elected not to include touch and taste due to the subjectivity and variability in how respondents might interpret these senses. Most people do not realize that the sense of taste is responsible for only a small set of oral sensations (e.g., sweet, sour, salty, bitter), often confuse the perceptions of taste and smell, and erroneously construe “taste” as primarily responsible for the enjoyment of food. Indeed, lacking awareness of the fact that olfaction is a primary driver of flavor could have led respondents to further undervalue their sense of smell in the present study. Interpretation of the sense of touch is also ambiguous, as it is often narrowly understood as relevant to pain and/or sexual pleasure, which would have biased respondents’ value of it accordingly. If this survey were administered with detailed explanations, all five senses should be included, and a complete and more accurate evaluation of olfaction’s place among the senses could be ascertained.

Another limitation stems from our participant sample. Respondents were recruited from exclusive east coast universities or were connected to friends and colleagues of the authors, and, as such, represented a narrow socio-demographic segment of North American society. It would be important now to administer this survey to a more diverse population and to translate the survey into different languages to determine how generalizable the findings are. This would further illuminate how individual differences mediate the value of the senses as well as various commodities. There was also a large imbalance in the number of male and female respondents. A more equal distribution of male and female respondents, as well as individuals who define themselves in non-binary gender terms, should have been included. Additionally, only three respondents in the GP and CS groups, respectively (*N* = 6), reported an olfactory impairment. As this represents less than 1.5% of all respondents, we do not believe it had an impact on the data. However, had a larger number of individuals with olfactory loss been tested, it might have altered the priority given to the other senses over smell. It is now of interest to administer this questionnaire to individuals with varying degrees of olfactory dysfunction.

A further limitation relevant to our sample was that we collected very minimal demographic information. Notably absent were any data on race, ethnicity, geography (location, rural-urban), to name but a few. Issues pertaining to geography may be especially germane for the GP group since they are from more widely dispersed locations than students from campuses that are only 60 miles apart; though since the data were collected during the COVID-19 pandemic when teaching at Boston College and Brown University was conducted remotely, many of the college students might have been living in their family home rather than on campus, thus representing a more varied geographic range. Lacking this information, however, curtails how much we can discern from our respondent samples and reiterates the importance of a direct examination of demographic factors in future research. 

Finally, despite an attempt to incorporate a range of commodities of varying psychological value and emotional meaningfulness, our commodity list was very limited (presenting only nine commodities from a potentially endless list) and was also unbalanced. For example, we included an approximately equal number of digital commodities (online shopping, social media, streaming services) and physical commodities ($10,000, hair, pet, little left toe), “phone” can be considered both a digital and a physical commodity, but only one aspirational commodity (dream vacation). There were also many inherent differences within categories for the various types of commodities as discussed above. If a longer survey were developed, it would be valuable to dive deeper into commodities representing a wider range of real-life and aspirational experiences as well as more commodities with intense emotional and social value. In future administrations of this survey, commodities could also be tailored for specific demographics; for instance, additional physical attribute commodities could be included if one were interested in gender differences and more material commodities if one were interested in socio-economic factors. Relatedly, although a discussion of the items with colleagues and a small pilot analysis was conducted during the survey development, we did not formally test the validity of our questionnaire prior to administration. We plan to use this questionnaire in future investigations which will help determine its validity and replicability, and we welcome any independent assessments.

## 5. Conclusions

The data from this survey study conducted with a large number of respondents have revealed a number of very important and impactful findings that have never before been detailed in this way. First, we have shown that in spite of current media attention and health coverage of the sense of smell, the sense of smell remains perceived as negligible in value and of trivial importance compared to the senses of hearing and vision. Second, we have shown the merit of contrasting different types of commodities for elucidating and evaluating the importance of the senses, and that money, cellphones, pets, and hair are particularly meaningful commodities. Third, our findings demonstrate that individual differences pertaining to gender and life-stage mediate the importance of the senses and the value of commodities we tested, with females and younger adults being especially likely to give up their sense of smell in order to keep various commodities. Finally, our survey results reveal previously unknown similarities in value between the senses of vision and hearing. Our findings now lead to many questions for future research, and an expanded survey comprising more commodities of varying value and contrasting all five senses will provide a comprehensive understanding of the value of the senses.

## Figures and Tables

**Table 1:** Subject Characteristics by General Population and College Student Groups.

	General Population	College Students
**TOTAL—*N*; %**	**219; 54%**	**188; 46%**
**Age, years—range** Age—Mean (Standard Deviation (SD)) ***	20–76 48 (±13)	18–65 22 (±9)
**Gender—*N*; %**		
Female Age—Mean (SD)	170; 78% 48 (±14)	143; 76% 22 (±8)
Male Age—Mean (SD)	47; 21% 48 (±10)	35; 19% 24 (±12)
Non-Binary	1; 0%	6; 3%
Prefer not to say	1; 0%	4; 2%
**Income Level—*N*; %**		
High **	48; 22%	60; 33%
Middle *	142; 65%	97; 53%
Low	25; 11%	19; 10%
Prefer not to say	4; 2%	12; 6%
**Sensory Impairment—*N*; %**		
None	156; 71%	142; 76%
Vision	52; 24%	39; 21%
Hearing	4; 2%	0; 0%
Smell	3; 1%	3; 2%
Vision + Hearing	2; 1%	2; 1%
Vision + Smell	2; 1%	0; 0%
Hearing + Smell	0; 0%	0; 0%
Hearing + Vision + Smell	0; 0%	2; 1%

Note: significance indicated by * *p* < 0.05, ** *p* < 0.01, *** *p* < 0.001.

**Table 2:** Degrees of freedom and Chi-square statistics (χ^2^) with significance for generalized linear mixed models composed of key variables and their interactions.

	Degrees of Freedom, χ^2^			
Variables	Model 1	Model 2	Model 3 ^¥^	Model 4 ^¥^
sense	2, 126.88 ***	2, 208.38 ***	2, 69.91 ***	2, 137.08 ***
commodity	8, 236.84 ***	8, 314.31 ***	6, 22.53 **	6, 33.57 ***
group	1, 12.35 ***	1, 20.80 ***	1, 11.61 ***	1, 17.70 ***
gender	1, 2.17	1, 6.70 **	1, 0.51	
sense*commodity	16, 59.08 ***	16, 55.33 ***	12, 34.04 ***	12, 29.49 **
commodity*group	8, 14.88		6, 7.06	
commodity*gender	8, 9.42		6, 5.36	
sense*group	2, 0.50		2, 1.62	
sense*gender	2, 0.15		2, 0.51	
Bayesian Information Criterion	4167	4009	2330	2193

Notes: ^¥^ indicates that **pet** and **hair** were not included in the *commodity* list (models 3 and 4); significance is indicated by, ** *p <* 0.01 and *** *p* < 0.001.

## Data Availability

Data available upon request.

## Appendix A

***Value of Our Senses Survey***

**Age**____

**Gender**: Female     Male     Non-Binary     Prefer not to say

**When thinking about you/your family’s financial status do you consider it to be:**

Low income     Middle income     High Income     Prefer not to say

**Without corrective devices do you consider yourself severely impaired in your (check any that apply):**

Hearing     Vision     Sense of Smell     None

__________________________________________________________________________

**Smell** = Your ability to smell

**Hearing** = Your ability to hear

**Vision =** Your ability to see

*Please answer each line item.*

	**What would you rather lose?**				
	My smell		My phone		
	My hearing		My phone		
	My vision		My phone		
	**What would you rather lose?**				
	My smell		$10,000		
	My hearing		$10,000		
	My vision		$10,000		
	**What would you rather lose?**				
	My smell		My favorite social media site		
	My hearing		My favorite social media site		
	My vision		My favorite social media site		
	**What would you rather lose?**				
	My smell		Online shopping		
	My hearing		Online shopping		
	My vision		Online shopping		
	**What would you rather lose?**				
	My smell		My favorite streaming service		
	My hearing		My favorite streaming service		
	My vision		My favorite streaming service		
	**What would you rather lose?**				
	My smell		My dream vacation		
	My hearing		My dream vacation		
	My vision		My dream vacation		
	**What would you rather lose?**				
	My smell		My pet		
	My hearing		My pet		
	My vision		My pet		
	**What would you rather lose?**				
	My smell		My hair		
	My hearing		My hair		
	My vision		My hair		
	**What would you rather lose?**				
	My smell		My left little toe		
	My hearing		My left little toe		
	My vision		My left little toe		
	**What does “anosmia” mean?**				
	Inability to hear words				
	Inability to see faces				
	Inability to taste				
	Inability to smell				
	**What would you least mind losing?**				
	My ability to smell		My ability to see		My ability to hear
